# Clonal dissemination and plasmid plasticity of KPC-3–producing *Klebsiella pneumoniae* ST512 during a hospital outbreak in Spain

**DOI:** 10.1007/s10096-026-05478-5

**Published:** 2026-04-15

**Authors:** María Riesgo-Magaña, Albert Moreno-Mingorance, Jessica Bueno, Sara Arnal, Elena Alvarado, Juan José González-López, Cristina Seral

**Affiliations:** 1 Hospital Clínico Universitario, Zaragoza, España; 2 Universidad de Zaragoza, Zaragoza, España; 3 Instituto de Investigación Sanitaria Aragón (IIS Aragon), Zaragoza, España; 4 Microbiology Research Group, Vall d’Hebron Institut de Recerca, Barcelona, Spain; 5 CIBER de Enfermedades Infecciosas (CIBERINFEC), Instituto de Salud Carlos III, Madrid, Spain; 6 Department of Genetics and Microbiology, Universitat Autònoma de Barcelona, Bellaterra, Spain; 7 Department of Clinical Microbiology, Hospital Universitari Vall d’Hebron, Barcelona, Spain

**Keywords:** *Klebsiella pneumoniae* ST512, KPC-3 carbapenemase, Hospital outbreak, Plasmid genomics, Whole-genome sequencing, IncFII(K), ColEST258

## Abstract

**Purpose:**

To describe the first outbreak of KPC-3-producing *Klebsiella pneumoniae* ST512 in Aragón, Spain, and characterize its clinical, microbiological, and genomic features, including plasmid dynamics, resistance mechanisms, and phylogenetic context.

**Methods:**

Between July 2022 and July 2024, 130 KPC-3-producing *K. pneumoniae* isolates were recovered from 33 patients during an outbreak at a tertiary-care hospital in Zaragoza. Antimicrobial susceptibility testing and whole-genome sequencing were performed. Phylogenomic (SNP and cgMLST) and plasmid analyses defined clonal relatedness and plasmid structures. Comparative genomics with 985 international ST512/KPC-3 genomes determined phylogeographic relationships.

**Results:**

Most cases (84.6%) were detected through active surveillance. All the isolates were resistant to β-lactams, ceftolozane/tazobactam, tobramycin and amikacin, while 64.4% remained susceptible to gentamicin. All the isolates were susceptible to cefiderocol, colistin, and tigecycline. One ceftazidime/avibactam-resistant isolate carrying *bla*_KPC−70_ emerged after prolonged therapy. Genomic analysis confirmed a clonal outbreak of *Klebsiella pneumoniae* ST512 (≤ 16 SNPs; ≤13 cgMLST allelic differences). Phylogenetic comparison showed that the isolates were genetically close to those from Italy and central Spain. All isolates carried *bla*_KPC−3_ within Tn*4401b*. Three *bla*_KPC−3_ plasmid structures were identified: an IncFII(K) plasmid (pHCUKPC3), a ColEST258 variant, and a novel cointegrate plasmid (pHCUKPC3co). The virulence-associated factors identified included yersiniabactin (ybt10/ICE*Kp4*), KL107 capsular type, and O2afg O-antigen.

**Conclusion:**

This study documents the wider dissemination of ST512/KPC-3 as a high-risk clone in Spain, characterized by persistence driven by clonal dissemination, selective pressure, and plasmid plasticity. Our findings highlight the need to integrate genomic and plasmidomic surveillance to anticipate resistance evolution and contain high-risk clones.

**Supplementary Information:**

The online version contains supplementary material available at 10.1007/s10096-026-05478-5.

## Introduction

KPC-producing *Klebsiella pneumoniae* (KPC-Kp) was first reported in the United States in 1996 [1] and has since spread worldwide, becoming the most prevalent carbapenemase [2]. Due to the limited therapeutic options and its association with infections characterized by high morbidity and mortality, the World Health Organization (WHO) included carbapenem-resistant *K. pneumoniae* on its priority pathogen list in 2017, a classification recently updated in 2024 [3].

Data from the European EuSCAPE Project [4] has demonstrated a significant increase in the prevalence of KPC-Kp in Europe, with higher incidence rates reported in countries such as Greece and Italy. Although OXA-48 remains the most prevalent and widely disseminated carbapenemase in Spain, KPC is the second most frequent, with two outbreaks caused by the high-risk clone ST512 having been reported [5, 6].

The genes encoding these carbapenemases are located on mobile genetic elements (MGEs), such as plasmids, transposons, and integrons, facilitating their horizontal dissemination among different bacterial species [7]. Furthermore, these MGEs are often associated with high-risk clones, notably ST258/512, contributing to nosocomial outbreaks with high transmission capacity and persistence in hospital settings [8].

Controlling outbreaks of KPC-Kp presents a substantial challenge due to its significant colonization and dissemination capabilities. Strict implementation of epidemiological surveillance measures in microbiology laboratories is essential for containment. In this context, whole-genome sequencing (WGS) has been increasingly recognized as an essential tool for early detection, molecular characterization, and monitoring of hospital outbreaks [9, 10].

The aim of this study was to describe the first outbreak of carbapenemase-producing *Enterobacteriaceae* reported in Aragón, an autonomous community in northeastern Spain, where no previous circulation of the ST512/KPC-3 clone had been documented. By combining epidemiological data with WGS, plasmid characterization, and phylogenomic analysis, we characterized the implicated isolates and provide new insights into the dissemination dynamics of these pathogens in the region.

## Methods

### Hospital setting and epidemiological investigation

Hospital Universitario Lozano Blesa is an 800-bed tertiary-care center in Zaragoza (Aragón, Spain) covering a population of 300,000. Before the outbreak, systematic screening for multidrug-resistant (MDR) bacteria carriage was routinely performed in the intensive care unit (ICU), including triple-site surveillance screening at ICU admission and weekly thereafter. Following the first detection of KPC-producing *Klebsiella pneumoniae* on July 15, 2022, from an abdominal abscess in a surgical patient, rectal screening of ward contacts was immediately initiated. As a result of this event, a risk-based, hospital-wide screening strategy for MDR bacteria was implemented. This targeted approach included rectal swabs for patients with predefined risk factors, such as prior hospitalization, recent exposure to broad-spectrum antibiotics, and/or transfer from facilities with high endemicity or ongoing outbreaks. Weekly multidisciplinary meetings (Preventive Medicine, Infectious Diseases, Microbiology, and clinical teams) guided outbreak control. Active surveillance included targeted screening, reinforcement of hand hygiene, and contact isolation. Environmental screening (faucets, drains, siphons) yielded no reservoir. Isolation was discontinued after three negative screenings. Clinical and microbiological data were retrieved from electronic medical records and descriptive analysis was performed.

### Case definition

An outbreak case was defined as any patient with clinical infection or colonization by KPC-Kp isolates exhibiting an identical antimicrobial susceptibility profile and confirmed KPC production using the NG-Test Carba 5 (NG-Biotech, Guipry-Messac, France).

### Microbiological methods

For surveillance screening, rectal swabs (hospital-wide) and triple-site surveillance in the intensive care unit (ICU) (rectal, pharyngeal, nasal) were cultured on chromID^®^ CARBA SMART agar (bioMérieux, Marcy-l’Étoile, France). Species identification was performed by Matrix-Assisted Laser Desorption/Ionization Time-Of-Flight Mass Spectrometry (MALDI-TOF MS) (Bruker Daltonics, Bremen, Germany). Antimicrobial susceptibility testing was performed with MicroScan WalkAway^®^ (Beckman Coulter, Brea, CA, USA) and interpreted using EUCAST v12.0, 2022 criteria. The minimum inhibitory concentrations (MICs) for ceftolozane/tazobactam (C/T), ceftazidime/avibactam (CAZ/AVI), meropenem/vaborbactam (MER/VAB), and imipenem/relebactam (IMI/REL) were determined by Etest^®^ (bioMérieux, Marcy-l’Étoile, France), and cefiderocol (Thermo Fisher Scientific, Waltham, MA, USA) by disk diffusion. Carbapenemase screening in clinical strains was initiated for MER (MIC > 0.125 mg/L). The presence and characterization of carbapenemases were determined using NG-Test Carba 5 (NG-Biotech, Guipry-Messac, France).

### Whole genome sequencing and analysis

One KPC-producing *K. pneumoniae* isolate per patient was further characterized by WGS. For this purpose, an axenic culture of the isolate was incubated for 18–24 h on COS agar medium (bioMérieux, Marcy-l’Étoile, France) with a 10 µg MER disk. DNA was extracted using the commercial magLEAD 12 cgDNA kit (Precision System Science, Japan) according to the manufacturer’s instructions. DNA quantification was performed with a Qubit 4.0 fluorometer (Thermo Fisher Scientific, USA). Illumina libraries were prepared using the Nextera XT DNA preparation kit (Illumina Inc., USA). Sequencing was conducted on the Illumina iSeq 100 platform, generating 2 × 150-bp paired-end reads (Illumina Inc., USA).

Reads were trimmed (Trimmomatic v0.39), assembled (Unicycler v0.5.0), and quality-checked (FastQC v.0.12.1 and QUAST v5.3.0). Genomes were typed and screened for resistance and virulence loci using Kleborate v2.0.4, Prokka v1.14.5, AMRFinder v3.12.8, and RGI v4.2.2.

Multilocus sequence typing (MLST) profiles were assigned and genomic distances inferred using Ridom^®^ SeqSphere+ v8.5.1 (cgMLST scheme, 2,358 loci) and complemented by core -single nucleotide polymorphism (SNP) analysis with Snippy v4.3.6. using ST512-K30BO as reference (GenBank accession no. NZ_CAJM00000000.2) [11]. Recombination was filtered (Gubbins v2.3.4), and phylogenies constructed by maximum likelihood. To assess fine-scale divergence within the outbreak cluster, we re-mapped outbreak isolates against the index case genome (Kpn_C1) and reconstructed a recombination-filtered SNP phylogeny.

### Clonality assessment and typing strategy

Clonality and transmission dynamics were inferred from WGS using genomic criteria. Isolates were considered clonally related when they differed by < 15 cgMLST alleles (2,358 loci) as previously described [12].

### Global phylogenomic context

To contextualize our isolates, a global core SNP maximum-likelihood phylogeny was generated (Supplementary Material) including 39 *K. pneumoniae* ST512 genomes from this study and 985 ST512/KPC-3 FASTQ files available from Pathogenwatch (accessed May 5, 2024). Reads were processed with Snippy using *K. pneumoniae* HS11286 (GenBank NC_016845.1) as reference, and recombination was removed with Gubbins.

Subsequently, a SNP-based phylotemporal tree was reconstructed with a Bayesian dated phylogeny using BactDating v1.1.2 after Gubbins analysis, focusing on the cluster formed by the outbreak strains and the most closely related strains retrieved from the literature, with Kpn_C1 the reference strain. Four molecular clock models (Poisson, Negative Binomial, Mixed Gamma, and Mixed Continuous Additive Relaxed Clock) were performed with a total of 10^7^ iterations each to ensure that the effective sample size of the inferred parameters exceeded 200 in the Markov chain Monte Carlo analysis. The model with the lowest deviance information criterion (Poisson model) was used for the final analysis. Significance of clock signal was tested by running the algorithm again with all sampling dates forced equal under the same conditions.

### Plasmid analysis

Five epidemiologically representative isolates were selected for plasmid characterization based on temporal and epidemiological diversity, as well as plasmid replicon profiles (PlasmidFinder; ≥95% coverage, ≥ 60% identity). Genomic DNA was extracted using the DNeasy^®^ Ultraclean Microbial Kit (Qiagen, Germany) and sequenced using MinION technology (Oxford Nanopore Technologies). Hybrid assemblies (short + long reads) were generated with Unicycler (v0.5.0) and annotated in Geneious Prime (v2024.0.5; Dotmatics). Plasmids carrying resistance determinants were compared with previously described sequences using blastn (National Center for Biotechnology Information, https://blast.ncbi.nlm.nih.gov) and the *progressiveMauve* algorithm implemented in Geneious Prime.

## Results

### Outbreak detection and transmission tracking

On July 15, 2022, the first case of KPC-Kp was identified in the General and Digestive Surgery Unit of Lozano Blesa University Hospital, Zaragoza. An extensive screening program was subsequently implemented, encompassing 480 patients from various wards and 367 from the ICU between July 2022 and September 2024.

**Fig. 1:**
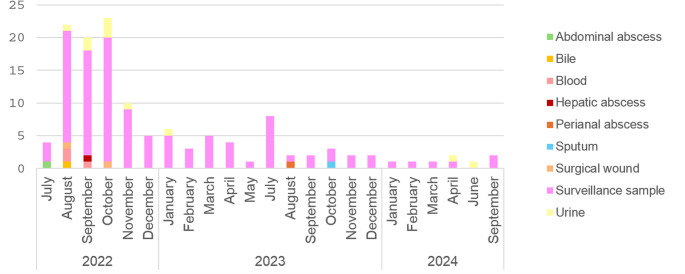
Temporal distribution of KPC-Kp isolates during the study period. A total of 130 isolates were represented, most being from surveillance samples (110/130), with the remainder obtained from clinical samples (20/130)

**Fig. 2:**
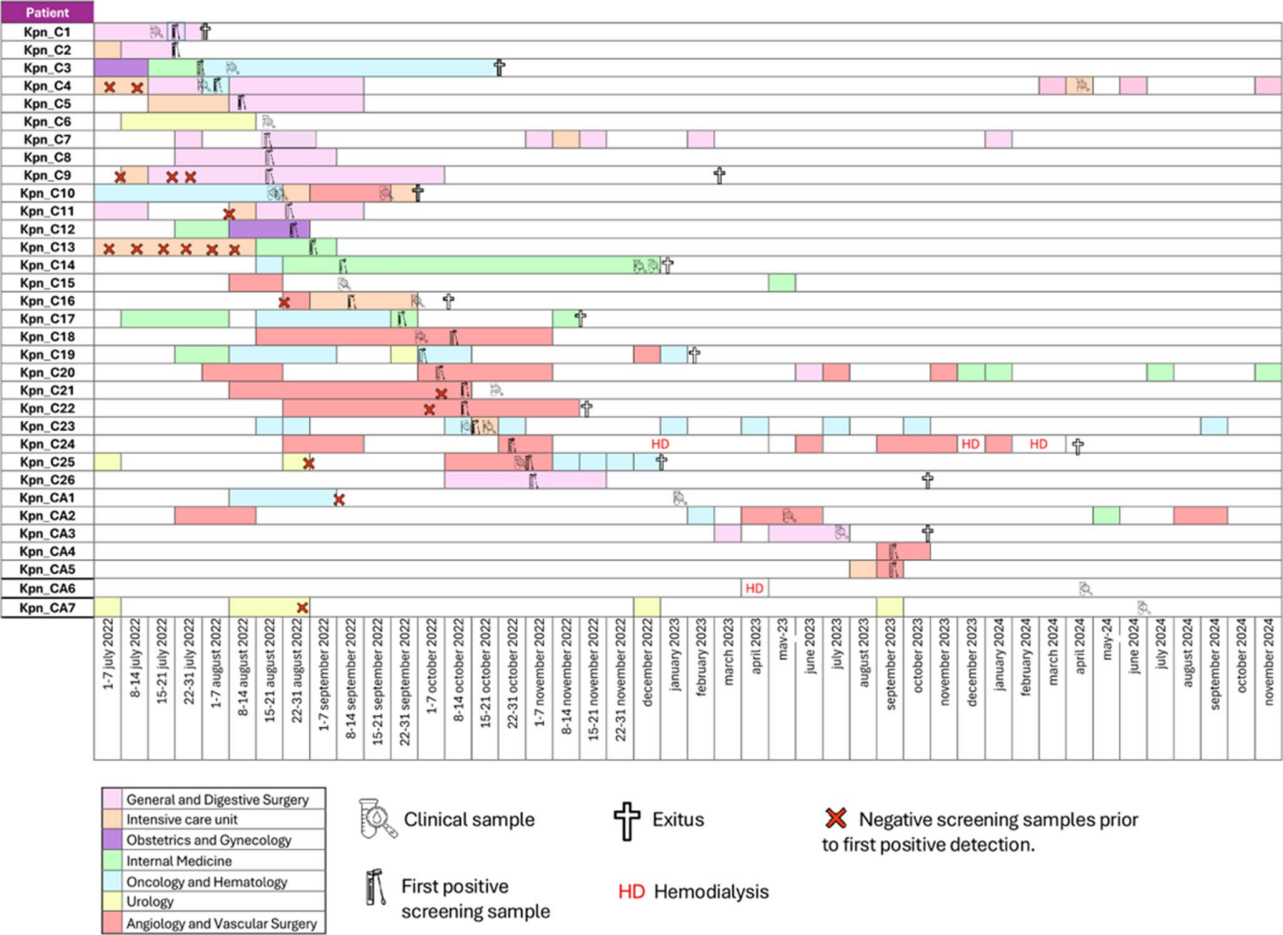
Temporal distribution of the 26 cases detected during the outbreak (Kpn_C1 to Kpn_C26) and the 7 additional related cases during 2023–2024 (Kpn_CA1 to Kpn_CA7). The timeline shows the dates when the first isolates were obtained either through active surveillance (screening) or from clinical samples and negative screening samples collected dates before the first positive screening sample was detected. Dates of death are also indicated for patients who died during follow-up. Hospital wards where patients were admitted are highlighted in different colors

A total of 130 KPC-Kp isolates were recovered, including 84.6% (110/130) from surveillance samples and 15.4% (20/130) from clinical samples, corresponding to 33 patients (Fig. 1). Among these, 48.5% (16/33) developed a clinical infection. Of the 33 patients, 14 (42.4%) had at least one negative screening result prior to their first positive detection, either before (from May onwards) or during the outbreak period. In four of them (Kp_C1, Kp_C2, Kp_C10, and Kp_C15), the preceding negative rectal swabs were collected between May and June and are therefore not shown in Fig. 2.

The most frequent infection source was urine (50%, 10/20) followed by blood cultures (15%, 3/20) and surgical wound exudates (10%, 2/20). Single cases were also detected in an abdominal abscess, perianal abscess, bile, sputum and non-surgical wound samples (5% each, 1/20). KPC-Kp was recovered from multiple sites in 3/16 (18,8%) patients: Kpn_C10 (blood, bile, liver abscess) and two patients with urine and sputum isolates (Kpn_C14 and Kpn_C23).

Most of the cases were reported from July to November 2022, (26 patients: Kpn_C1-Kpn_C26) (Fig. 1). The majority were linked to General and Digestive Surgery (*n* = 9), Angiology and Vascular Surgery (*n* = 6), and Internal Medicine (*n* = 4), with sporadic cases in Oncology-Hematology (*n* = 3) and single cases in Obstetrics and Gynecology and in the ICU. Two additional cases were diagnosed after hospital discharge.

Between 2023 and 2024, seven additional patients colonized or infected with KPC-Kp were identified (Kpn_CA1-Kpn_CA7). Three (Kpn_CA1, Kpn_CA2, Kpn_CA7) had been hospitalized from July to November 2022 but remained undetected at that time. The remaining four (Kpn_CA3-Kpn_CA6) were admitted afterwards; of these, two (Kpn_CA4 and Kpn_CA5) overlapped spatiotemporally with patient Kpn_C24, while one (Kpn_CA6) coincided with Kpn_C24 during hemodialysis. As shown in Fig. 2, five of these additional cases were identified in 2023 (one each in January, May, and July, and two in September), and two more were detected in 2024 (April and July); the latter marking the final case and the end of the outbreak.

### Clinical and epidemiological data

The main epidemiological characteristics of the patients, along with antibiotic treatments and clinical outcomes, are summarized in Table 1.

**Table 1:** Clinical and epidemiological characteristics of patients with KPC-Kp infection/colonization (*n* = 33). CHT/RT chemotherapy and/or radiotherapy; IQR: interquartile range; ICU: intensive care unit

Clinical and epidemiological characteristics	Number of patients (%)
Demographics and others	
Patients	33
Age, Median (IQR)	69 years (62.5–76.5)
Gender	Female (45.5), Male (54.5)
Previously hospitalization under 6 months	28 (84.8)
ICU hospitalization during outbreak	14 (42.4)
Underlying disease	
Dementia	1 (3.0)
Hypertension	22 (66.7)
Diabetes mellitus	10 (30.3)
Heart failure	5 (15.2)
Chronic renal insufficiency	15 (45.5)
Peripheral vascular disease	8 (24.2)
Cerebrovascular disease	1 (3.0)
Chronic pulmonary disease	6 (18.2)
Cancer	11 (33.3)
Organ transplant	2 (6.1)
Hemodialysis	3 (9.1)
Invasive procedures	
Urinary manipulation	24 (72.7)
Mechanical ventilation	5 (15.2)
Vascular catheter	33 (100)
Drains	21 (63.6)
Endoscopy	13 (39.4)
Nasogastric tube	5 (15.2)
Digestive surgery	18 (54.5)
Vascular surgery	9 (27.3)
Other surgeries	10 (30.3)
Treatments administered in the 30 days prior to KPC detection	
CHT/RT	7 (21.2)
Monoclonal antibodies	6 (18.2)
ß-lactams	30 (90.9)
Amoxicillin/clavulanate	18 (54.5)
Ceftazidime	3 (9.1)
Cefazolin	8 (54.5)
Cefixime	1 (3.0)
Cefuroxime	3 (9.1)
Ceftriaxone	15 (45.5)
Cefepime	3 (9.1)
Piperacillin/tazobactam	18 (54.5)
Carbapenems	21 (63.6)
Imipenem	18 (54.5)
Meropenem	1 (3.0)
Meropenem and Ertapenem	2 (6.1)
Ceftolozane/tazobactam	4 (12.1)
Ceftazidime/avibactam	3 (9.1)
Meropenem/vaborbactam	1 (3.0)
Other antibiotic	4 (12.1)
Mortality	
14 days	2 (6.1)
30 days	3 (9.1)
> 30 days	9 (27.3)

### Antibimicrobial susceptibility

Antimicrobial susceptibility testing was performed in 45 isolates, comprising 20 from clinical samples and 25 representing the first surveillance isolate from each colonized patient, providing representative data and minimizing data duplication (Supplementary Material).

All isolates were resistant to classical β-lactams, carbapenems, and C/T, but remained susceptible to MER/VAB, IMI/REL, and cefiderocol.

Regarding aminoglycosides, all the isolates were resistant to tobramycin and amikacin, whereas 64.4% (29/45) remained susceptible to gentamicin. Susceptibility to trimethoprim/sulfamethoxazole was observed in 17.8% (8/45) of the isolates. All isolates were susceptible to tigecycline and colistin, while none were susceptible to fluoroquinolones or nitrofurantoin.

On September 19, one month after a bacteremia episode, a *K. pneumoniae* isolate resistant to CAZ/AVI (MIC > 256 mg/L) was recovered (isolate Kp19). This strain exhibited reduced susceptibility to cefepime (MIC 4 mg/L), aztreonam (MIC ≤ 1 mg/L), and carbapenems: ertapenem (MIC ≤ 0.12 mg/L), IMI (MIC ≤ 1 mg/L), and MER (MIC 1 mg/L). Carbapenemase detection by lateral flow immunochromatography was negative; however, the presence of the *bla*_KPC_ gene was confirmed by polymerase chain reaction (Xpert^®^ Carba-R, Cepheid, USA).

### Global context and phylogeny analysis

**Fig. 3:**
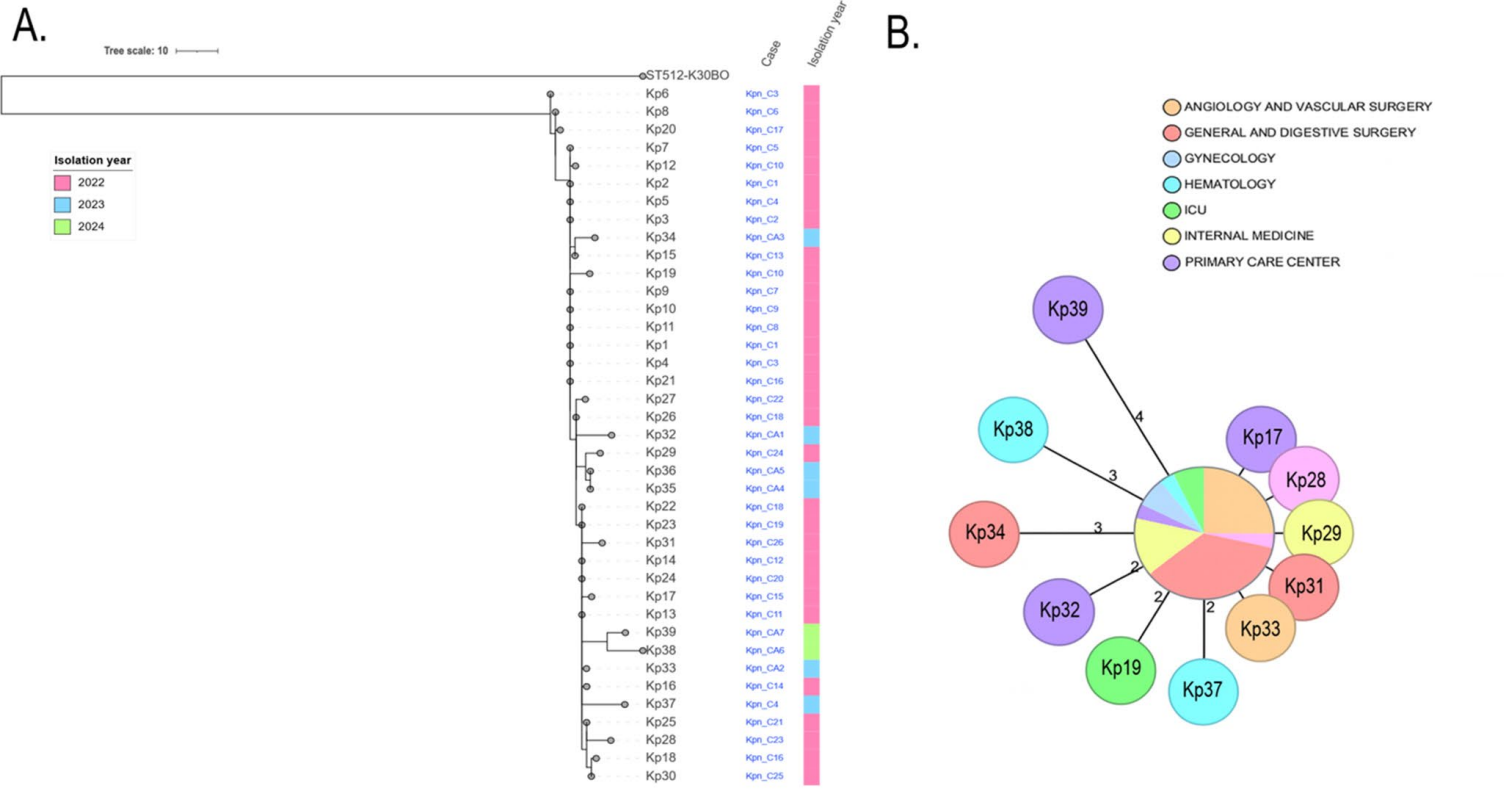
**A** Maximum-likelihood phylogenetic tree of *Klebsiella pneumoniae* isolates from core-genome SNP alignments and isolation year. The tree was rooted with the reference strain *K. pneumoniae* ST512-K30BO, and branch support values were estimated with 1,000 bootstrap replicates. The scale bar represents the number of nucleotide substitutions per site. **B** Minimum spanning tree showing allelic relationships among *K. pneumoniae* isolates. Each node represents a genome or genome cluster, with edge labels indicating the number of allelic mismatches. Colors correspond to the wards where the isolates were recovered. See the SNP distance matrix and the cgMLST locus-encoded matrix in Supplementary Material

To optimize resources, at least one isolate per patient was sequenced, and when available, both the first clinical and the first surveillance isolate were included, given the restricted number of frozen samples preserved and the high cost of large-scale genomic analysis. A total of 39 isolates were sequenced (27 from surveillance samples and 12 from clinical samples) and incorporated into downstream analysis (Fig. 3).

MLST classified all the isolates as sequence type (ST) 512. cgMLST analysis revealed a central cluster comprising 28 genetically indistinguishable isolates (0 allelic differences) distributed across multiple hospital services, indicating widespread dissemination of the core outbreak clone. Additional isolates were located at the periphery of the minimum spanning tree, differing by only 1–4 alleles from the cluster. Overall, pairwise cgMLST distances ranged from 0 to 13 alleles (median 2, interquartile range [IQR] 1–4).

In addition, whole-genome SNP comparisons provided confirmation of higher-resolution consistent with the cgMLST structure. Distances ranged from 0 to 16 SNPs (median 3, IQR 0–5). Nearly one third of the isolate pairs (28.3%) were genetically indistinguishable (0 SNPs), and the vast majority were within 5 SNPs (78.1%) or 10 SNPs (97.2%), supporting the predominance of a single clonal lineage driving the outbreak. One large 0-SNP cluster grouped 21 isolates across General and Digestive Surgery (*n* = 9), Vascular Surgery (*n* = 5), Internal Medicine (*n* = 3), Hematology (*n* = 2), and the ICU (*n* = 2), illustrating both intra-service transmission and inter-ward dissemination. At ≤ 10 SNPs, 36 isolates collapsed into a single connected cluster.

**Fig. 4:**
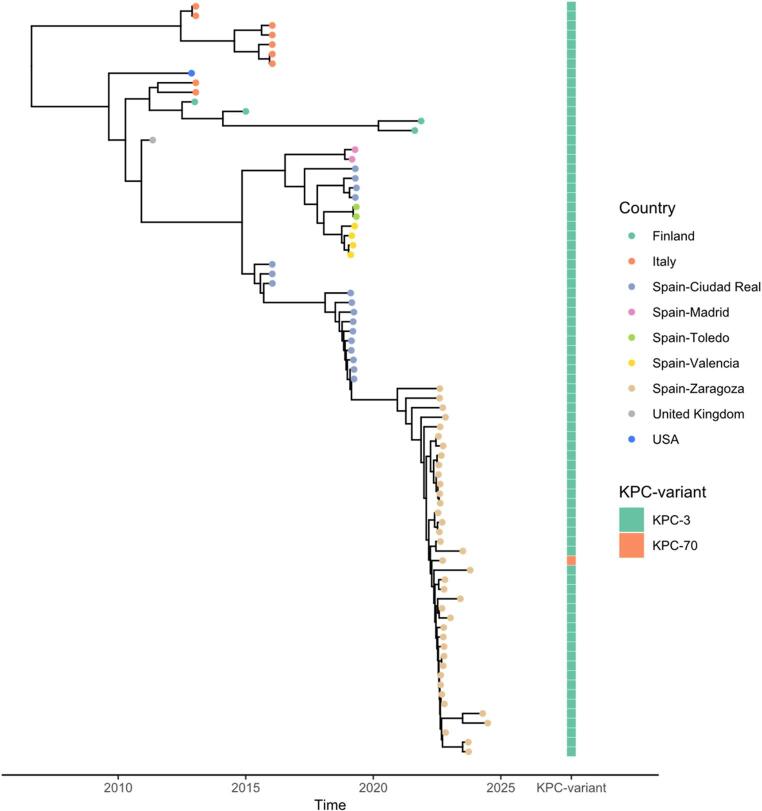
Bayesian phylogenetic reconstruction of 79 KPC-Kp ST512 isolates, 39 genomes from the outbreak strains of this study and 40 genomes with genomic data available in Pathogenwatch. The country or city of origin for each isolate is represented with colored dots at the end of the tree branches

When our outbreak genomes were compared with isolates previously sequenced in other studies, the outbreak isolates formed a tight cluster within 79 closely related ST512 genomes forming a clade. A Bayesian phylogenetic tree of this clade is shown in Fig. 4. The majority of isolates originated from other regions of Spain, with the most closely related genomes being obtained in Ciudad Real (minimum distance of 32 SNPs from the outbreak genomes). Other genomes were obtained from Italy, Finland, and a single isolate each from the United Kingdom and the United States (Supplementary Material).

### Antimicrobial resistance and virulence determinants

The resistance-associated genes consistently identified in all outbreak isolates included *bla*_KPC-3_, *bla*_SHV-11_, *aac(6′)-Ib10*, *aadA2*, *sul1* and *fosA5*. A Kp19 isolate exhibiting reduced carbapenem susceptibility harbored the same set of resistance determinants but WGS confirmed the presence of the *bla*_KPC-70_, a derivative of *bla*_KPC-3_ carrying two single amino acid substitutions: D179Y and T263A. This isolate was recovered from a liver abscess in patient Kpn_C10. This patient had previously been colonized and subsequently developed an intra-abdominal infection caused by KPC-Kp and extensively drug-resistant *Pseudomonas aeruginosa*, which progressed to a mixed bloodstream infection. The patient had received C/T (1 g every 8 h, one day) followed by CAZ/AVI (2 g every 8 h, one day). After detection of the CAZ/AVI-resistant isolate, antimicrobial therapy was switched to MER/VAB (1 g every 8 h, nine days). Despite targeted therapy, the patient ultimately died.

Chromosomal mutations were detected in the quinolone resistance determining region (QRDR) (*gyrA* S83I and *parC* S80I), alongside the *bla*_SHV-11_ variant (35Q) in all the isolates. Porin analysis revealed a truncated *ompK35* (25% of full length) due to frameshift mutations c.5dupG (p.Gly3fs) and c.122dupG (p.Glu42fs), as well as a point mutation in *ompK36 *(D135DGD).

The yersiniabactin locus ST78 (ybt10 subtype) was located within the integrative conjugative element ICE*Kp4*. Additionally, siderophore systems were identified and included enterobactin (*entA-F*). No genes encoding aerobactin, colibactin or hypermucoviscosity-associated regulators (*rmpA*, *rmpA2*) were detected. Capsular typing based on *wzi*154 corresponded to KL107, while O-antigen typing revealed the O1/O2v2 locus, consistent with the O2*afg* serotype.

### Plasmidome analysis

**Fig. 5:**
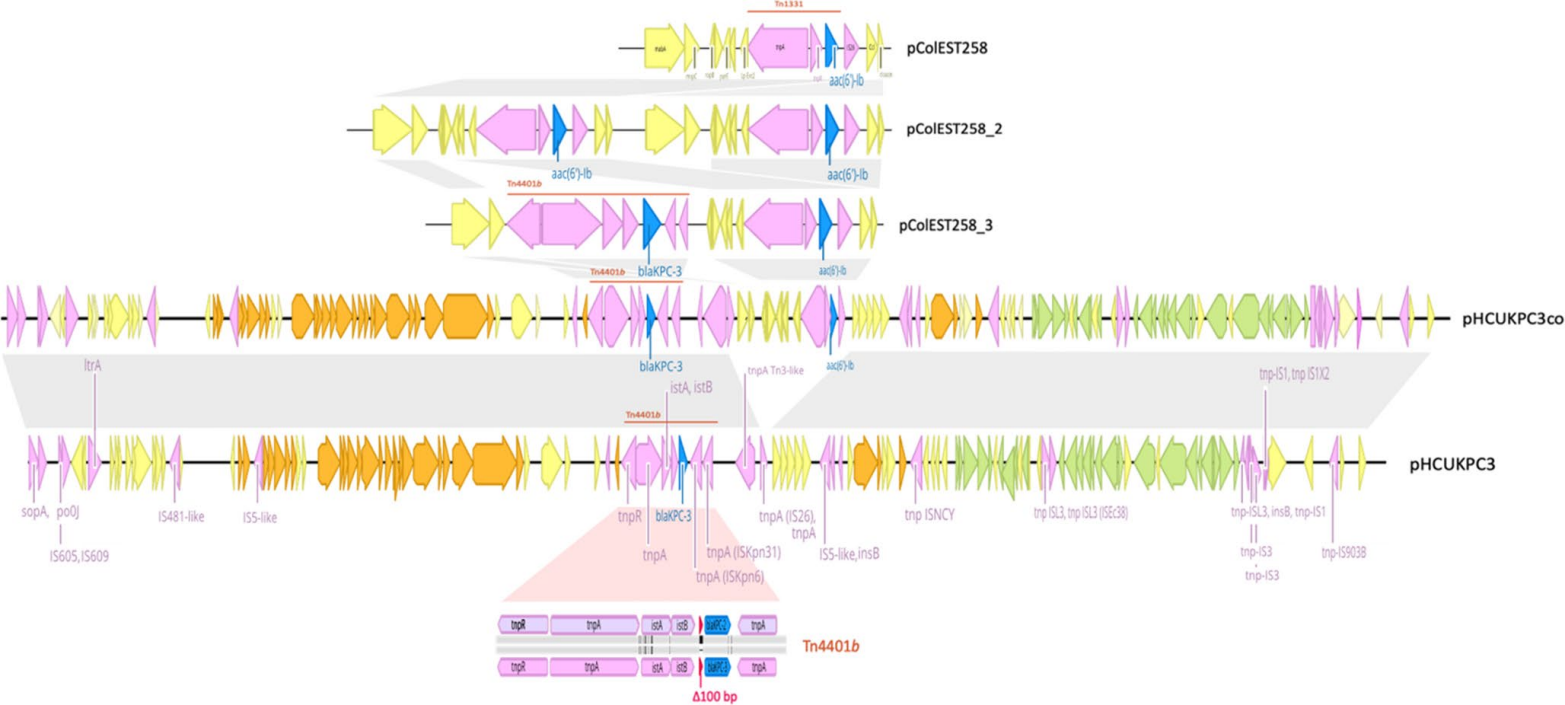
Schematic representation of the plasmid structures identified in the outbreak isolates, including pHCUKPC3, pHCUKPC3co, ColEST258, ColEST258_2, ColEST258_3 and Tn*4401b*. Regions with 100% nucleotide identity are shown in light grey. ORFs are indicated by arrows and colored according to functional categories: antibiotic resistance genes (blue), metal resistance genes (light green), conjugation tra locus (orange), transposon-related genes (pink) and plasmid scaffold /accessory genes (yellow)

Plasmid characterization was performed in the five selected isolates shown in Table 2. Selection was based on their temporal and epidemiological heterogeneity, as well as their distinct replicon profiles. The genetic distance between these plasmids ranged from 0 to 16 SNPs, and five distinct plasmids were identified (Fig. 5): pHCUKPC3, pHCUKPC3co, ColEST258, ColST258_2, and ColST258_3.

**Table 2:** Plasmids identified in each isolate. The table includes the date of isolation, isolate reference, and the plasmids detected

Isolation date	Patient	Sample (isolate reference)	Identified plasmids
15 July 2022	Kpn_C1	Abdominal abscess (Kp1)	pHCUKPC3co
10 August 2022	Kpn_C6	Urine (Kp8)	pHCUKPC3, ColEST258 and ColEST258_3
17 August 2022	Kpn_C10	Blood (Kp12)	pHCUKPC3co and ColEST258_2
19 September 2022	Kpn_C10	Hepatic abscess (Kp19)	pHCUKPC3co
18 October 2023	Kpn_C23	Sputum (Kp37)	pHCUKPC3co and ColEST258_2

pHCUKPC3 was an IncFII(K) plasmid (161,964 bp), which contained 169 open reading frames (ORFs) related to replication, plasmid transmission, and resistance to antibiotics and heavy metals. It carried *bla*_KPC-3_ within transposon Tn*4401b* (10,006 bp), as confirmed by alignment with the reference Tn*4401b* sequence (GenBank accession no. EU176013.1), showing 100% query coverage and 98.9% identity [13]. Comparative analysis revealed 100% query coverage and 99.98% identity with plasmid pBK32179 (GenBank accession no. JX430448) [14]. A second plasmid, p1512-dfrA (GenBank accession no. MF918373.1), also showed high similarity (85% query coverage, 99.78% identity).

pHCUKPC3co (175,600 bp), was a cointegrate plasmid derived from the fusion of pHCUKPC3 and ColEST258, encompassing 190 ORFs involved in replication, conjugation, and resistance to antibiotics and heavy metals. To our knowledge, this plasmid structure has not been previously described.

Small ColE1-*like* plasmids were identified. The first, ColEST258 (13,605 bp; GenBank accession no. JN247853), originally described by García-Fernández et al. [15], carried the *aac(6’)-Ib10* resistance gene within the Tn*1331* transposon. A related plasmid, ColEST258_2 (27,272 bp), consisting of two copies of ColEST258, was detected in some isolates. Finally, the third *bla*_KPC-3_ carrying structure identified was designated pColEST258_3 (23,547 bp), harboring both Tn*4401b* and Tn*1331* with the *aac(6’)-Ib10* gene.

## Discussion

This study reports and molecularly characterizes the first documented outbreak of KPC-3–producing *Klebsiella pneumoniae* ST512 in Aragón, Spain. The initial outbreak involved 26 patients admitted to the University Hospital Lozano Blesa (Zaragoza) over a five-month period (July to November 2022). Following the implementation of outbreak containment measures, no new cases were detected until January 2023. During 2023 and 2024, seven additional epidemiologically related cases were identified.

The outbreak was clustered in surgical units (General and Digestive Surgery, Vascular Surgery) but also extended to other departments, illustrating both intra- and inter-ward transmission. Epidemiological investigation revealed a staggered temporal distribution, which peaked in August 2022 with 10 cases, consistent with sustained nosocomial transmission across overlapping chains. Although initial environmental sampling did not reveal a reservoir, systematic sampling was not performed.

Most affected patients in our cohort were older than 60 years, had prior hospitalizations (84.8%), and had frequently received carbapenems (63.6%). Prior carbapenem exposure has been repeatedly identified as the strongest driver for infections by carbapenem-resistant *K. pneumoniae* [16], in line with our findings. Advanced age, invasive procedures, and ICU admission have also been linked to progression from colonization to infection [17]. In our series, 48.5% of patients developed infections, and the bacteremia rate among colonized patients was 9.1%.

This figure was lower than that reported by Cano et al. [18], who reported an incidence of subsequent bacteremia of 52.4% among patients colonized with KPC-3–producing *K. pneumoniae* ST512 during the first outbreak described in Spain. Conversely, our rate was higher than in the multicenter cohort by Giannella et al. [19] in Italy, where 7.8% of patients colonized with KPC-Kp developed bacteremia across participating hospitals.

All isolates were resistant to conventional β-lactams and C/T but remained susceptible to MER/VAB, IMI/REL, and cefiderocol, in line with European multicenter data on KPC-3 producers [20]. The detection of the KPC-70 variant after prolonged CAZ/AVI therapy illustrates classical adaptive evolution under selective pressure [21]. KPC variants with Ω-loop substitutions may regain partial carbapenem susceptibility, potentially compromising CAZ/AVI efficacy and impacting treatment decisions [22]. Beyond these epidemiological observations, the genomic background of the circulating clone provides additional context for interpreting our findings. We performed WGS which revealed that all isolates belonged to ST512. *K. pneumoniae* ST512 is a single-locus variant of ST258, both within clonal complex 258 (CC258) [11], a high-risk lineage associated with global healthcare dissemination and antimicrobial resistance [23, 24]. ST512 is strongly linked to KPC-3 production [25] but may also harbor other β-lactamases, including *bla*_TEM_, *bla*_CTX-M_, or *bla*_OXA-48-like_ [26–28], and rare co-production of NDM-1 has been documented [29]. The ST512/KPC-3 clone exhibited a KL107-wzi154/O1-O2v2 profile with yersiniabactin and fimbrial adhesins, a pattern repeatedly reported in Spain, Italy, and Poland [4, 5, 30] and characteristic of CC258 lineages [31], supporting persistence through intestinal colonization and environmental survival as key drivers of nosocomial dissemination rather than hypervirulence.

In Europe, Italy and Greece are the main epicenters of ST512/KPC-3 endemicity [8, 32], characterized by sustained transmission in hospitals and long-term care facilities. In contrast, in Spain, KPC-Kp remains largely outbreak-driven rather than endemic [33], with well-documented exceptions. The first ST258/KPC-3 outbreak was in Granada between 2015 and 2017 [34]; it was rapidly controlled and did not progress to endemic circulation. In contrast, the first ST512/KPC-3 outbreak was reported in Córdoba in 2012 and was linked to a patient transferred from Italy [5]. It rapidly spread through ICU and surgical wards, affecting 95 patients over two months, ultimately evolving into endemicity. Between 2016 and 2019, Ciudad Real experienced sustained interhospital dissemination of ST512/KPC-3 involving two hospitals and 156 KPC-Kp isolates [6]. Similar to Córdoba, this outbreak was not contained and evolved into endemic circulation, driven by patient transfers between wards and chronic-care facilities. Epidemiologically, our four-month outbreak with subsequent sporadic detections suggests silent persistence in carriers, representing an intermediate scenario between rapid control and long-term endemicity.

In our outbreak, phylogenomic reconstruction revealed a homogeneous central cluster of 28 cgMLST-identical isolates (0 allelic differences), with minor microevolutionary variants differing by only 1–4 alleles. SNP analysis confirmed these findings, grouping 36 isolates within a single cluster (≤ 10 SNPs). When linked to epidemiological data (Fig. 2), the phylogenomic findings provided insight into transmission dynamics across hospital wards. The outbreak appeared to emerge in the General and Digestive Surgery ward, but by July, isolates were also detected in Internal Medicine and Oncology-Hematology. Circulation in Vascular Surgery was not apparent until September, although cases Kpn_C15 and Kpn_C16, both linked to this ward in August, suggest earlier unnoticed transmission, consistent with the spatial proximity of these units on the second floor of the hospital. The epidemiological connection to Urology, where patient Kpn_C6 had been admitted in July, remains less clear, possibly reflecting transmission from an undetected colonized patient or healthcare staff movement.

Additional cases were detected months later, underscoring the role of undetected carriers and prolonged colonization in sustaining clonal circulation. Among these, Kpn_CA4 and Kpn_CA5 were indistinguishable from Kpn_C24 and overlapped temporally in the hospital, suggesting direct transmission, while Kpn_CA6 shared hemodialysis sessions with Kpn_C24, a plausible scenario for clonal spread. In contrast, Kpn_CA1, Kpn_CA2, and Kpn_CA7 had been hospitalized during the outbreak but were not detected at that time.

Although phylogenomics has transformed outbreak investigations, it cannot be used in isolation to define nosocomial transmission events. Temporal and epidemiological data remain essential to contextualize genomic findings. In our study, the high concordance between cgMLST and SNP analyses, with most isolates differing by ≤ 10 SNPs and ≤ 4 alleles, supports a monoclonal introduction followed by intra-hospital dissemination with limited microevolution. However, there is no universally accepted SNP threshold to define transmission events. Previous studies have proposed cutoffs ranging from 10 to 20 SNPs to support epidemiological linkage in *K. pneumoniae* [35, 36]. These findings underscore the need to integrate phylogenomics with detailed clinical and epidemiological information to accurately reconstruct transmission dynamics.

SNP analysis clustered our isolates within the group of predominantly Spanish genomes, showing the closest genetic relatedness to strains from Ciudad Real [6], Valencia and Madrid [33], which constitute the most representative nodes of this cluster. The cluster also encompassed Italian ST512/KPC-3 genomes [4], together with additional isolates from Finland, the United Kingdom and the United States [37–39], supporting the broad international dissemination of this high-risk clone and its well-established Mediterranean epidemiological linkage. This close phylogenetic relationship suggests a shared transmission network linking multiple Spanish regions and European settings. Several, non-mutually exclusive routes could account for this pattern, including patient transfer between institutions or via an intermediate healthcare facility, introduction from a widely circulating ST512 sublineage in Spain, and/or the involvement of an undetected index case. Notably, prior admissions or inter-hospital transfers to other institutions could not be reliably ascertained from our electronic health record, and therefore these routes cannot be formally evaluated in this dataset.

On the other hand, plasmid analysis revealed heterogeneous configurations across outbreak isolates, highlighting the dynamic nature of the plasmid-borne resistome. Previous studies have shown that ST512 *K. pneumoniae* exhibits marked plasmid plasticity, resulting in substantial intra-outbreak variability in plasmid content. This dynamic behavior has been documented both within individual patients, with the coexistence of KPC-producing and non-KPC-producing ST512 strains during a single colonization episode [40], and at the outbreak level, where plasmid loss and recombination generate distinct plasmid profiles among epidemiologically linked isolates [41]. Comparative studies across different regions further reveal structural plasmid diversity, including hybrid and fusion plasmids carrying multiple resistance and virulence determinants [42–44]. These findings complicate outbreak tracking and underscore the need for comprehensive genomic surveillance to fully characterize transmission events and resistance dynamics.

Initially, the worldwide dissemination of *bla*_KPC−3_ in *K. pneumoniae* ST512 was attributed to the pKpQIL plasmid, an epidemic IncFII(K) replicon carrying *bla*_KPC−3_, first described in Israel and later in Italy, and strongly associated with CC258 lineages (ST258 and ST512) [45]. In the present study, we identified different plasmid structures. First, pHCUKPC3, an IncFII(K) plasmid of 161.9 kb carrying *bla*_KPC-3_ within Tn*4401b*, was highly similar to pBK32179 [13] and p1512-dfrA (Beijing, China; unpublished). In contrast, comparative analysis revealed notable differences between our plasmid and the reference pKpQIL revealing only 29.5% pairwise identity, reflecting the presence of additional metal resistance genes in pHCUKPC3 that are absent in pKpQIL (Supplementary Fig. 1). This confirms the remarkable conservation of IncFII(K) backbones across continents and over time.

A particularly novel finding was the identification of a cointegrate plasmid, pHCUKPC3co, which combined elements of pHCUKPC3 (IncFII(K)) and ColEST258 (ColE1-*like*). We hypothesize that cointegration between the IncFII(K) and ColE1-*like* backbones was most plausibly IS*26*-mediated, given the presence of an IS*6*/IS*26*-family transposase and the lack of extended sequence homology between the two replicons. Experimental studies have demonstrated that IS*26* promotes replicative transposition and recombination between dispersed copies, generating stable mosaic plasmids that can persist under selective pressure [46]. In vivo evidence further supports this mechanism: Jiang et al. reported IS*26*-mediated acquisition of *bla*_KPC-2_ in a clinical ST11-K64 isolate, confirming that such events can occur within a single patient [47]. Finally, Chavda et al. provided early evidence of *bla*_KPC_-harboring cointegrates with horizontal transfer capacity in *Escherichia coli*, confirming that such mosaic plasmids can arise in vivo and disseminate resistance determinants across species [48]. Notably, BLAST analysis did not identify any previously described plasmid with a similar structure in NCBI, indicating that, to our knowledge, pHCUKPC3co represents the first cointegrate of its kind.

In addition to being part of the IncFII(K)–ColE1-*like* cointegrate, ColE1-*like* replicons were also found. These small replicons carried *aac(6′)-Ib10* within Tn*1331* in all three variants identified in our cohort, a configuration repeatedly documented in CC258 lineages and frequently coexisting with IncFII plasmids [49]. Notably, ColEST258_2, with duplication of ColEST258, was almost identical to plasmid p4 from the Córdoba ST512 outbreak [5], supporting the role of ColE1-*like* plasmids as hotspots for gene amplification. The most striking finding was ColEST258_3 in Kpn_C6, where Tn*4401* carrying blaKPC-3 was inserted into a ColE1-*like* plasmid already harboring Tn*1331*. While a nested arrangement of Tn*4401* within Tn*1331* has been previously described in the IncI2 plasmid pBK15692 [50], in ColEST258_3, Tn*1331* and Tn*4401* were both present as independent modules within the same ColE1-*like* replicon, rather than nested. All *bla*_KPC−3_ alleles in our isolates were embedded within Tn*4401*, consistent with its well-established role as the main driver of *bla*_KPC−_ mobilization [51]. In our cohort, the element corresponded to the Tn*4401b* isoform, characterized by a 100-bp deletion upstream of *bla*_KPC_ (between *ist*B and the gene), a configuration classically associated with *bla*_KPC−2_ in *K. pneumoniae* [52]. Nevertheless, this isoform has also been linked to *bla*_KPC−3_, including in an outbreak in North Carolina driven by *E. cloacae* and *K. pneumoniae* [53], and in a collection of Portuguese clinical isolates of *K. pneumoniae* ST147 and ST15 [54].

These findings illustrate how plasmid modularity and plasticity shape the evolutionary dynamics of ST512/KPC-3. The stable cointegration of IncFII(K) and ColE1-*like* replicons ensures long-term maintenance of resistance determinants, while ColE1-*like* plasmids act as integration hubs for multiple mobile elements, accelerating the accumulation and reshuffling of *bla*_KPC_ alleles. This adaptive architecture enables rapid diversification within a conserved backbone, supporting both clonal persistence and epidemic spread.

### Limitations

This study has some limitations, including the restricted number of isolates subjected to long-read sequencing, the lack of a comprehensive environmental investigation, and the inherent constraints of single-center data. Moreover, it was not possible to determine whether any of the patients had previously been hospitalized in another autonomous community, which would further support the hypothesis of interregional transmission.

## Conclusion

The integration of genomic and plasmidomic approaches has provided a robust and representative view of the outbreak of KPC-3-producing *Klebsiella pneumoniae* ST512 documented in Aragón. This is the first documented outbreak detected in this Spanish region, and it has been characterized by rapid intra-hospital dissemination, persistence beyond the acute phase, and microevolution within a single clonal lineage. Resistance profiling revealed limited therapeutic options and the emergence of *bla*_KPC-70_ under selective pressure. Plasmid analysis identified conserved IncFII(K) backbones, highly plastic ColE1-like replicons, and a novel cointegrate plasmid, illustrating the modularity of the plasmidome as a driver of ST512 success. These findings highlight the importance of integrating WGS and plasmidomic approaches into routine surveillance programs to anticipate resistance evolution and contain the spread of high-risk clones.

## Supplementary Information

Below is the link to the electronic supplementary material.

Supplementary Material 1.

Supplementary Material 2.

## Data Availability

All raw reads were submitted to NCBI under BioProject PRJNA1297134.
