# Supplementary figures and images for: Clonal dissemination and plasmid plasticity of KPC-3–producing Klebsiella pneumoniae ST512 during a hospital outbreak in Spain
Source: Eur J Clin Microbiol Infect Dis. 2026 Apr 15;45(8):2303–16. doi: 10.1007/s10096-026-05478-5 (PMC13428716; doi:10.1007/s10096-026-05478-5)

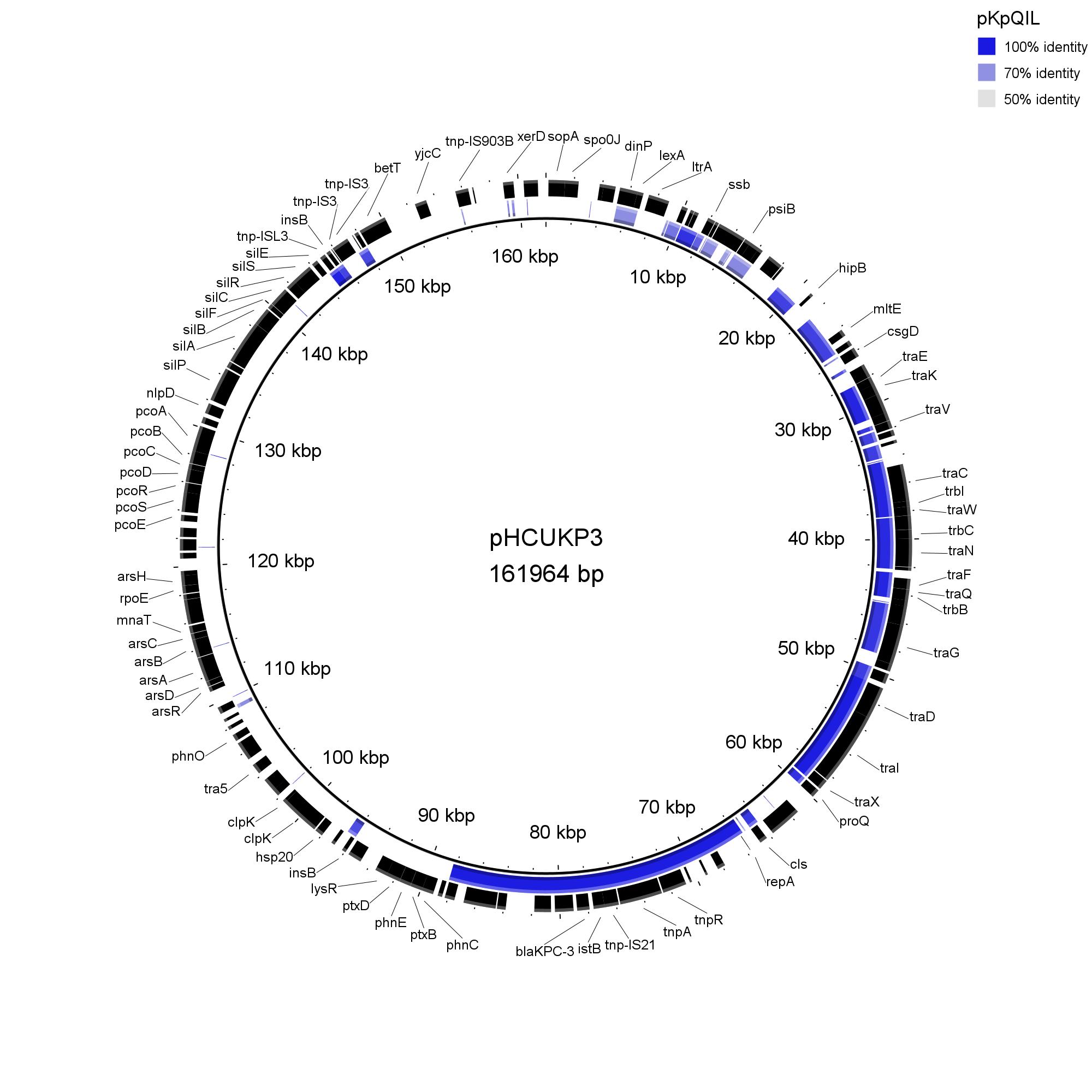

Supplement: Supplementary file 2 — Supplementary Material 2. [file 10096_2026_5478_MOESM2_ESM.jpg]
